# Predictors of excellent early outcome after total hip arthroplasty

**DOI:** 10.1186/1749-799X-7-13

**Published:** 2012-03-25

**Authors:** George H Smith, Simon Johnson, James A Ballantyne, Edward Dunstan, Ivan J Brenkel

**Affiliations:** 1 Department of Trauma and Orthopaedics, Queen Margaret Hospital, Dunfermline, KY12 0SU Scotland, UK

**Keywords:** Excellent, Outcome, Total hip Arthroplasty, predictors

## Abstract

**Background:**

Not all patients gain the same degree of improvement from total hip replacement and the reasons for this are not clear. Many investigators have assessed predictors of general outcome after hip surgery. This study is unique in its quest for the predictors of the best possible early outcome.

**Methods:**

We prospectively collected data on 1318 total hip replacements. Prior to surgery patient characteristics, demographics and co-morbidities were documented. Hip function and general health was assessed using the Harris Hip score (HHS) and the Short-Form 36 respectively. The HHS was repeated at three years. We took a maximal HHS of 100 to represent an excellent outcome (102 patients). Multiple logistic regression analysis was used to identify independent predictors of excellent outcome.

**Results:**

The two strongest predictive factors in achieving an excellent result were young age and a high pre-operative HHS (p = 0.001).

**Conclusions:**

It was the young and those less disabled from their arthritis that excelled at three years. When making a decision about the timing of hip arthroplasty surgery it is important to take into account the age and pre-operative function of the patient. Whether these patients continue to excel however will be the basis of future research.

## Disclaimer

This study did not receive public or private fundings nor do any authors have a proprietary interest.

## Introduction

Total hip arthroplasty (THA) has been shown to provide both significant improvements in the quality of life to patients with hip arthritis [1] but also an excellent cost per Quality-Adjusted Life Year (QALY) gain of half (€6710) that seen in total knee arthroplasty (€13995) [2]. Not all patients however gain the same degree of improvement and the reasons for this are not clear. Many investigators have assessed predictors of outcome after hip surgery [3-7]. This prospective study is unique in its quest for the predictors of the best possible early outcome.

## Materials and methods

Between 1998 and 2004 a dedicated audit nurse collected data prospectively on 1318 consecutive unilateral THA. Ethics committee approval was obtained.

Data collected pre-operatively included patient age, sex, body mass index (BMI), smoking status, medical co-morbidities (presence of hypertension, coronary heart disease and diabetes), any use of non-steroidal anti-inflammatory drugs (NSAIDS) or aspirin, ASA grade (American Society of Anaesthesiologists), pre-operative haemoglobin (Hb) and level of social deprivation (based on the patient's home post-code).

All of the operations were primary procedures and involved cemented acetabular and cemented femoral prostheses. All patients received prophylactic intravenous cephalosporins and the surgery was conducted in a theatre with laminar flow. They were all performed, or supervised, by a consultant orthopaedic surgeon using the approach most familiar to them. Cementing technique, rehabilitation and follow up were identical for each patient.

Outcome was assessed using two different assessment measures. The first was a joint specific measure - The Harris Hip Score (HHS) and the second was a general health questionnaire - Short Form 36 (SF-36).

The HHS is an extended hip function evaluation, which assesses the patient's perception of pain, function, ability to undertake activities and range of hip motion. The score ranges from 0 to 100, with higher scores indicating increased perceived success and satisfaction [8]. We chose a post-operative HHS of 100 to indicate a patient's perception of excellent outcome.

The SF-36 is a 36-item questionnaire that produces scores in eight domains relating to the patient's quality of life. These are physical functioning, role limitation due to physical problems, bodily pain, general health perception, emotional vitality, social functioning, role limitation due to emotional problems and mental health.

Data was collected pre-operatively and at three years of follow-up. Previous work has shown that HHSs plateau post-total hip replacement at around 18 months [1]. At 3 years therefore we would not expect our patients to see much more in the way of improvement.

### Statistics

All data was held in a regional arthroplasty database and recorded in Microsoft Excel format. Data was transferred to SPSS statistical software where the association between a HHS of 100 was tested by chi-squared or t-tests for each factor separately. For factors that gave significant results in these analyses, multiple logistic regression was then used to test for the effect of each factor adjusted for the others. A p value of < 0.05 was considered significant and < 0.001 highly significant.

## Results

We reviewed 1682 unilateral THAs performed within the six-year recording period. Data was incomplete for 364 patients (111 patients died before the three-year follow up and 253 did not have complete data). This left 1318 patients to enter analysis. We defined an excellent outcome as a patient having a maximum HHS of 100. In our study 102 patients (7.7%) had a HHS of 100 at three years. The average age of all the study patients was 68.5 (SD 9.9) years. The average age for the patients with a HHS of 100 was 62.0 (SD 9.9).

Highly significant independent predictors (p values < 0.001) of a HHS of 100 were; male sex, young age, low ASA grade, low body mass index, high pre-operative HHS, low deprivation levels and the absence of a history of hypertension or coronary disease. All but 2 of the 8 SF-36 variables (Role Emotional and Mental Health) were highly significant (p < 0.001). (Tables 1, 2, 3).

**Table 1:** Demographic Variables -significance of predicting excellent outcome at three years

Variable	P Value
Age	< 0.001
Sex	0.039
Body Mass Index	0.010
Smoking Status	0.76
Deprivation Level	0.007

**Table 2:** Pre-operative Variables -significance of predicting excellent outcome at three years

Variable	P Value
Hypertension	0.006
Diabetes	0.27
Coronary Disease	0.005
Aspirin	0.29
NSAIDS	0.018
ASA Grade	< 0.001
Pre-operative Hb	0.09

**Table 3:** Pre-operative Assessment Scores -significance of predicting excellent outcome at three years

Variable	P Value
Pre-operative HHS	< 0.001
SF-36 Physical Function	< 0.001
SF-36 Role Physical	< 0.001
SF-36 Body Pain	< 0.001
SF-36 General Health	< 0.001
SF-36 Emotional Vitality	< 0.001
SF-36 Social Functioning	< 0.001
SF-36 Role Emotional	0.09
SF-36 Mental Health	0.049

Multiple logistic regression analysis identified a young age (p = < 0.001) and a high pre-operative HHS (p = 0.001) as the two most significant associations with an excellent outcome (Table 4).

**Table 4:** Multiple Logistic Regression Analysis for the two most significant independent variables - significance of predicting excellent outcome at three years

Variable	P Value
High Pre-operative HHS	0.001
Young Age	< 0.001

## Discussion

The British Orthopaedic Association state the indications for THA are severe pain and disability, with accompanying radiological changes at the hip in patients where non-operative treatment has failed or is futile [9]. It has previously been tradition for arthroplasty surgery to be delayed for as long as the patient can tolerate. This is probably a consequence of the paucity of historical long-term follow-up for joint replacements. More recent research has questioned this belief with younger patients appearing to achieve better outcomes than their aged counterparts [3,10]. Fortin et al [11] suggested performing arthroplasty surgery earlier in the course of functional decline may be associated with better outcome. Lingard et al [12] demonstrated marked functional limitation, severe pain and a low mental health score before total knee arthroplasty were predictors of worse outcome. Patients with poor pre-operative walking distance are less likely to gain the same benefits from THA [13].

Of the 1318 patients enrolled in this study the two most powerful predictors of an excellent outcome at three years (HHS of 100) were a high pre-operative HHS and a young age at the time of surgery.

The HHS is an extended hip function evaluation, which assesses the patient's perception of pain, function, ability to undertake activities and range of hip motion. The score ranges from 0 to 100, with higher scores indicating perceived success and satisfaction. Marchetti et al [14] suggested that a HHS of 90-100 indicates an excellent result, 80-90 a good result, 70-80 a fair result and less than 70 a poor result.

The HHS was initially designed to assess the outcome of arthroplasty on traumatic arthritis after hip dislocation and acetabular fracture [8]. It has subsequently been shown to be both a sensitive and specific marker of hip function. It is more responsive than walking speed, pain and sub-scales of function of the SF-36 in patients with OA [15]. Soderman and Malchau [16] confirmed the HHS as having high validity and reliability when compared with other outcome scoring systems (Western Ontario and McMaster University Osteoarthritis Index (WOMAC) and the Medical Outcomes Study 36- Item Short-Form Health Survey questionnaires). A weakness of the HHS however is that it assumes a concordance of the views between the clinician and patient. Rothwell et al [17] illustrated how patients and clinicians can differ in their subjective importance of different elements in quality of life assessment.

Whether a maximum score of 100 out of 100 truly represents excellence is debatable. Johanson et al [4] noted final outcome score assessments do not take into account clinical improvement from the base line. This could be seen as a weakness in this study.

A standard was required; hence the arbitrary figure of 100 was selected. This produced 7.7% (n = 102) of patients who reported the best possible score from surgery. This itself is of significance when related to the patient's consent process. Only one patient in thirteen will express no complaints whatsoever at the three-year follow-up. As improvement in patient satisfaction is rare beyond eighteen months [1] any grievances are liable to remain.

It was the younger patients and those less disabled from their arthritis who excelled in this study. This is invaluable information to use during the consent process. At three years follow-up patients can expect the best possible result from their hip arthroplasty when they are relatively young and were less disabled from their arthritis. This would imply surgery earlier in the disease may give better early results. What is not clear however is the long term results of hip arthropasty at a young age. Hilmarsson et al [18] demonstrated in the Swedish hip registry 10-year survivorship of only 64-67% for hip replacements in patients under 55 years. Callaghan [19] saw a 29% revision rate at 20-25 years after THR when less than 50 years old. This would imply that although young patients may get an excellent early result the overall lifespan of the replacement is likely to be less. The increased levels of activity and the subsequent wear seen in the younger age group may explain this. In total knee replacements however the converse is true. In a prospective study of 622 knees Brenkel and Elson [20] demonstrated a young age as an independent predictor of pain from a knee replacement at five years. The authors speculated this could have been due to the development of a pain syndrome secondary to multiple previous operations. This is an entity not normally seen in hip arthroplasty surgery.

The overall improvement for hip replacements in the young may not be as great. In a health-status questionnaire MacWilliam et al [21] demonstrated for each 10-point increase in the preoperative score patients could expect at least a 6-point decrease in postoperative improvement.

In summary, when making a decision about the timing of hip arthroplasty surgery it is important to consider the age and pre operative function of the patient. These are strong predictive factors in achieving an early excellent result at three years. Whether these patients continue to excel however is not known and will be the foundations of future research.

## Competing interests

The authors declare that they have no competing interests.

## Authors' contributions

GS wrote the manuscript. SJ collated the data, set up the data analysis and helped draft the manuscript. JAB reviewed the manuscript and was involved throughout with patient recruitment. ED conceived the original idea, was involved in patient recruitment and proof read the manuscript. IJB set up the data collection, participated throughout in patient recruitment and proof read the manuscript.
